# *Plasmodium* centrin *Pb*CEN-4 localizes to the putative MTOC and is dispensable for malaria parasite proliferation

**DOI:** 10.1242/bio.036822

**Published:** 2018-12-12

**Authors:** Magali Roques, Rebecca R. Stanway, Edward I. Rea, Robert Markus, Declan Brady, Anthony A. Holder, David S. Guttery, Rita Tewari

**Affiliations:** 1 School of Life Sciences, Queens Medical Centre, University of Nottingham, Nottingham NG7 2UH, UK; 2 Institute of Cell Biology, University of Bern, Bern 3012, Switzerland; 3 The Francis Crick Institute, London NW1 1AT, UK; 4 The Leicester Cancer Research Centre, College of Life Sciences, University of Leicester, Leicester LE2 7LX, UK

**Keywords:** Asexual replication, Centrin, Centriolar plaque, Closed mitosis, Microtubule-organizing centre (MTOC), *Plasmodium*, Spindle pole body

## Abstract

Centrins are calmodulin-like phosphoproteins present in the centrosome and play an active role in the duplication, separation and organization of centrosomal structures such as the microtubule-organizing centre (MTOC) during mitosis. They are also major components of the basal body of flagella and cilia. In *Plasmodium* spp., the parasite that causes malaria, mitosis is closed during asexual replication and the MTOC is embedded within the intact nuclear membrane. The MTOC has been named the centriolar plaque and is similar to the spindle pole body in yeast. In all phases of asexual replication, repeated rounds of nuclear division precede cell division. However, our knowledge of the location and function of centrins during this process is limited. Previous studies have identified four putative centrins in the human parasite *Plasmodium* *falciparum.* We report here the cellular localization of an alveolate-specific centrin (*Pb*CEN-4) during the atypical cell division of asexual replicative stages, using live cell imaging with the rodent malaria parasite *P. berghei* as a model system. We show that this centrin forms a multi-protein complex with other centrins, but is dispensable for parasite proliferation.

## Abstract (precis)

**Summary:** This study examines the localization of malaria parasite centrin *Pb*CEN4 at the parasite MTOC during closed endomitosis and shows it to be dispensable for proliferation.

## INTRODUCTION

*Plasmodium* species, the causative agents of malaria, undergo two types of atypical closed mitotic cell division during their complex life cycles. The first is during schizogony within hepatocytes and erythrocytes in the vertebrate host and sporogony within the oocyst attached to the mosquito midgut. The second is an endoreduplicative process during the sexual stage, where a rapid threefold DNA replication within 8–10 min results in the formation of eight male gametes within the mosquito midgut (Sinden, 1991; Arnot et al., 2011; Gerald et al., 2011; Guttery et al., 2012). Closed mitosis in the *Plasmodium* parasite is characterized by multiple rounds of DNA replication and asynchronous nuclear division, without dissolution of the nuclear membrane. This karyokinesis proceeds in the absence of concomitant cytokinesis, forming a multinucleate syncytium, and cytokinesis only occurs once the multiple rounds of nuclear division are complete. In erythrocytes, the final round of DNA replication and segregation is synchronous and coordinated with final daughter merozoite assembly (Arnot et al., 2011).

In several eukaryotes, particularly humans, plants and yeast, the mechanisms controlling nuclear and cell division, and the molecules involved are well studied. Progression through mitosis requires many proteins, including centrins, cyclins, protein kinases, phosphatases and the microtubule organizing centre (MTOC) (Sluder, 2005; Harashima et al., 2013). Previous studies in *Plasmodium* have identified key genes whose products regulate the different phases of the life cycle and the distinctive transitions between them. In particular, systematic gene knockout screens have highlighted the importance of several protein kinases and phosphatases as likely essential for erythrocytic schizogony and for sexual stage development (Tewari et al., 2010; Solyakov et al., 2011; Guttery et al., 2014; Gomes et al., 2015). Most recently, *Pf*crk4 and *Pf*cyclin1 have been shown to be essential for *P. falciparum* asexual blood stage proliferation (Ganter et al., 2017; Robbins et al., 2017) with *Pf*cyclin1 involved in cytokinesis (Robbins et al., 2017). However many cell cycle regulators are absent from the parasite, including classical cyclins, various anaphase-promoting complex (APC) molecules, CDC25 and CDC14 (Guttery et al., 2014; Roques et al., 2015; Wall et al., 2018).

The MTOC is the centrosome in mammalian cells, the spindle pole body (SPB) embedded in the nuclear membrane in yeast, and the basal body in flagellated and ciliated cells (Zhang and He, 2012; Seybold and Schiebel, 2013; Bornens and Gönczy, 2014). In *Plasmodium,* as in budding yeast, mitosis is closed and occurs without dissolution of the nuclear envelope. The putative MTOC (as it will be named here) resembles the yeast SPB, serving as the anchor for the mitotic spindles, and has also been referred to as the ‘centriolar plaque’, despite clear centriolar structures not having been observed (Arnot et al., 2011; Gerald et al., 2011). During the cell cycle, the MTOC has to be duplicated and separated to allow correct progression through mitosis, and centrin proteins are among the main orchestrators of these events (Azimzadeh and Bornens, 2007; Mardin and Schiebel, 2012).

Centrins (also known as caltractrins) belong to a family of calmodulin-like calcium-binding phosphoproteins containing four EF-hand domains (Wright et al., 1985; Salisbury, 1995; Zhang and He, 2012). Centrin was initially identified at the centrioles in the green alga *Chlamydomonas reinhardtii* (Wright et al., 1985), and is associated with the MTOC (Bornens and Gönczy, 2014) in the pericentriolar material (PCM), as well as at the distal lumen of centrioles (Wright et al., 1985; Baron et al., 1992; Paoletti et al., 1996). Four centrins have been described in mammals (Huang et al., 1988) and grouped into those belonging to a subfamily related to budding yeast CDC31 (centrin 3) or those with most homology to the *C. reinhardtii* centrin (centrin 1, 2 and 4) (Middendorp et al., 1997, 2000).

There have been some functional studies of centrins in protozoa. In the ciliate *Tetrahymena,* CEN-1 and -2 function in basal body orientation, maintenance, and separation (Vonderfecht et al., 2012). In the flagellated *Kinetoplastida* group, e.g. *Leishmania donovani*, CEN-1 is involved in the duplication of basal bodies in amastigote stages*,* and in *Trypanosoma brucei,* deletion of *cen-1*, *-2* and *-3* affected organelle segregation and potentially inhibited cytokinesis (Selvapandiyan et al., 2012). In Apicomplexa, *Toxoplasma gondii* has three centrins, which have been localized to an MTOC-like structure during cell division (Hu et al., 2006; Hu, 2008). Four centrin-like proteins have been identified in *P. falciparum* (*Pf*CEN-1 to -4) and phylogenetic analysis showed that CEN-2 and -4 are members of an alveolate-specific subgroup (Mahajan et al., 2008). Transcription and protein expression profiles demonstrated that these centrins are differentially expressed at different stages of the life cycle (Hall et al., 2005; Mahajan et al., 2008). By microscopy and co-localization with specific MTOC markers, *Pf*CEN-2 and *Pf*CEN-3 were observed to be associated with the putative MTOC, close to the nucleus in the sporozoite and in asexual stages for *Pf*CEN-2 and *Pf*CEN-3, respectively (Mahajan et al., 2008).

Here, using live cell imaging, we characterize the dynamics of an alveolate-specific *Plasmodium* centrin (CEN-4) in the rodent malaria parasite *P. berghei,* to examine atypical mitotic division in all three phases of asexual replication. *Pb*CEN-4 shows a distinct temporal profile – it is cytoplasmic preceding replication, but then locates to discrete foci that correspond to the putative MTOC structure during nuclear division. CEN-4 forms a part of a multi-centrin protein complex. However, the deletion of *cen-4* has no effect on parasite proliferation and this redundancy is not due to compensation by the increased expression of one of the other centrin genes.

## RESULTS

### Live cell imaging reveals a temporal profile of CEN-4 at discrete foci corresponding to the putative MTOC during asexual replication

To study the dynamics and function of the four centrins identified in *Plasmodium*, we used gene tagging and reverse genetics approaches. Of the four centrin genes, *cen-1*, *-2*, *-3* and *-4*, in the *P. berghei* genome (Nagamune and Sibley, 2006; Mahajan et al., 2008) three: *cen-1* (PBANKA_0206300), *cen-2* (PBANKA_1310400) and *cen-3* (PBANKA_0511800) were refractory to both deletion and modification by different C-terminal tagging strategies; single homologous recombination to generate an endogenously GFP-, mCherry- or HA-tagged version. This suggests that these proteins are likely essential for blood stage parasite growth.

This result is consistent with the output of a large-scale *P. berghei* functional gene screen, in which *cen-1* and *cen-2* were described as essential genes (Bushell et al., 2017), although no data are available for *cen-3* or *cen-4.* Here, we focus on *cen-4* (PBANKA_0941400), which we were able both to express as a protein with a C-terminal GFP-tag in the parasite and to delete to study its role during parasite development.

To investigate the subcellular localization of CEN-4, we generated *P. berghei* parasites expressing the protein with a C-terminal GFP tag, by inserting the GFP coding sequence by single homologous recombination at the 3′-end of the *cen-4* coding sequence (Fig. S1A,B). Diagnostic PCR confirmed successful integration (Fig. S1C). Western blot analysis of parasite extracts using an anti-GFP antibody showed that the CEN4-GFP fusion protein has the expected size (47 kDa) in comparison to the 29 kDa unfused GFP constitutively expressed by the WT-GFPcon 507 cl1 line, referred to here as the wild-type (WT) parasite (Fig. S1D).

**Fig. 1.**
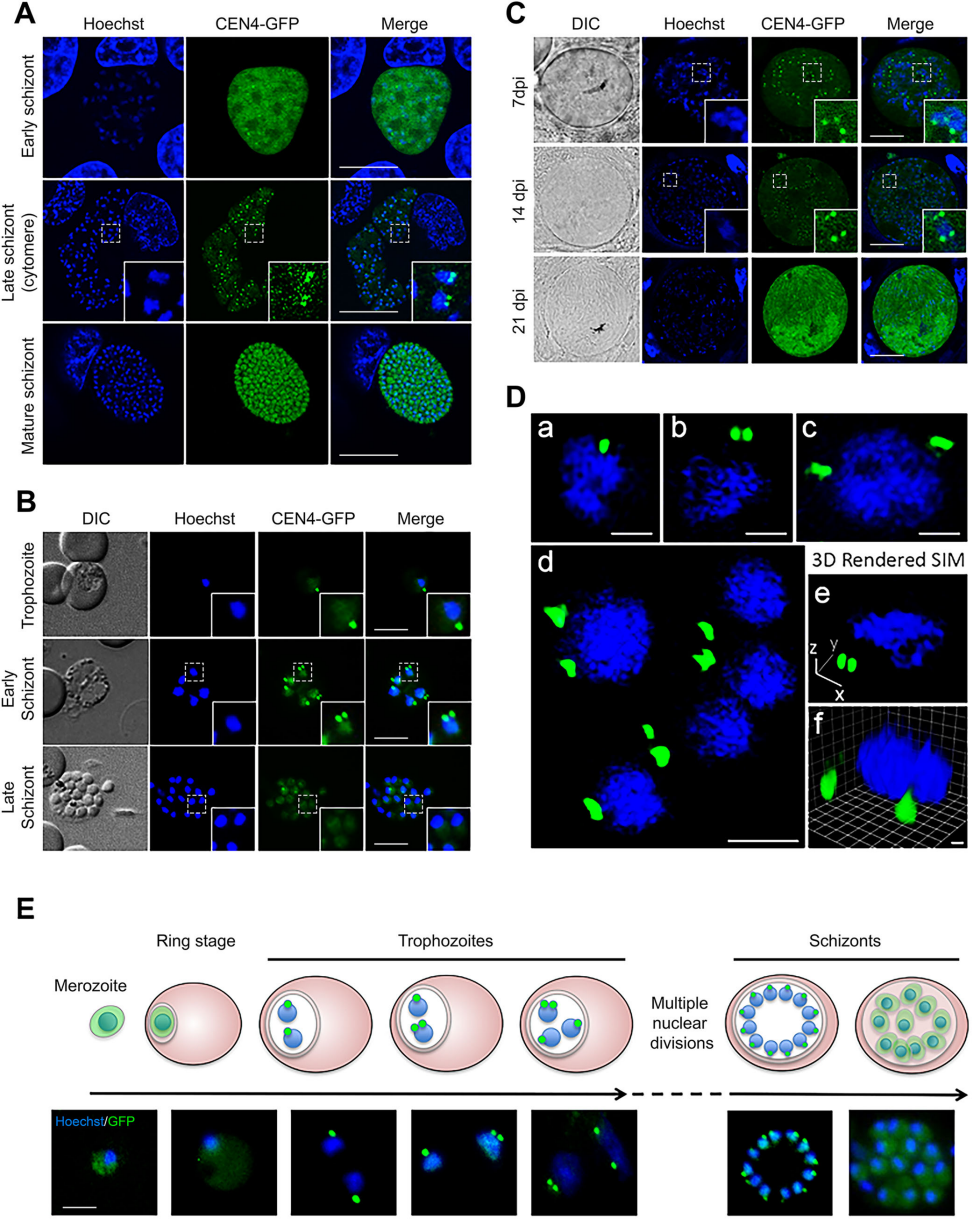
**A centrosomal location of CEN-4 during schizogony.** (A) Live images of the liver stage parasite at three time points: early schizont (nuclear replication has begun, top row, scale bar: 10 µm), late schizont (cytomere stage, middle row, scale bar: 20 µm) and mature schizont (bottom row, scale bar: 20 µm). (B) Live images of the blood stage parasite at three time points: trophozoite (prior to nuclear replication, top row), early schizont (nuclei are replicating, middle row) and late schizont (formation of mature merozoites between 18–24 h post invasion, bottom row). Scale bars: 5 µm. (C) Live images of sporogony in the mosquito oocyst: 7 dpi (top row), 14 dpi (middle row) and 21 dpi (bottom row). Scale bars: 5 µm. (D) 2D-maximum intensity projections (a–d) and 3D rendered (e–f) super resolution microscopy images from fixed asexual blood stage parasites. (a) One CEN-4 focus. (b) Duplicated CEN-4 foci. (c) Two segregated CEN-4 foci. (d) Asynchronicity of nuclear division in a single blood stage schizont, all focal planes are visible in Movie 1. (e,f) 3D models of a single schizont with divided putative MTOC, displayed here from a side view or tilted 45°, full rotation videos are visible in Movies 2–5. Scale bars in a–c: 0.5 µm; d: 1 µm; f: 0.2 µm. (E) Schematic diagram representing closed mitosis in schizogony. A CEN4-GFP-expressing merozoite (Merozoite) (cytosolic CEN4-GFP in green) invades a red blood cell and following ring (Ring stage) and trophozoite stages, schizogony commences with nuclear division (Immature schizonts) and CEN-4 is relocated to foci. In one parasite, division of one nucleus is accompanied by CEN-4 splitting into two foci close to each other. These foci then segregate, and nuclear division continues asynchronously until the last synchronous round of division (immature schizonts). During this time, CEN-4 remains at foci but becomes cytosolic once merozoites are fully formed within the red blood cell (mature schizonts). Corresponding representative images are given below. Scale bar: 5 µm. The panels A and C are images from confocal microscopy and panels B and E are from epifluorescence microscopy. Images from the panel D are from super resolution microscopy. The green channel is CEN4-GFP fluorescence, the blue channel is Hoechst-stained DNA and the DIC image is provided for the blood and mosquito stages. Merge is a merged image of green and blue channels. Insets are selected areas of the panels at higher magnification.

The temporal dynamics of centrin in the CEN4-GFP transgenic line were studied at three asexual replicative stages: liver and red blood cell stages in the vertebrate host and the oocyst stage in the mosquito vector. In early liver stage schizonts, CEN4-GFP was located throughout the cytosol but also displayed discrete foci (Fig. 1A top row), whilst showing a clear localization at distinct perinuclear foci in blood stage trophozoites, and in developing oocysts 7 days post-infection (dpi) in mosquitoes [Fig. 1B,C (top row),Da, respectively]. During early blood stage schizogony, as the nuclei begin to replicate and divide, the CEN4-GFP foci duplicate, resulting in two foci remaining close together and adjacent to each nucleus [Fig. 1B (middle row),Db]. At this time, the DNA in the nucleus is replicating but the nucleus only divides once the two centrin foci have separated to opposite sides of the nucleus (Fig. 1A–C second panel,Dc). Immediately prior to cytokinesis, one final round of synchronous nuclear division occurs in the schizont. Within the liver and mosquito stages, it was difficult to visualize the early stages of asexual replication, with a clear CEN4-GFP signal only being visible after a few rounds of nuclear replication (Fig. 1A,C middle row, respectively). At the cytomere stage in the liver, the CEN-4 signal remains as a dot in close proximity to each nucleus. At completion of daughter cell formation in all three phases of schizogony/sporogony, the CEN4-GFP signal becomes cytoplasmic as daughter cells are newly formed (Fig. 1A–C, bottom row). Super resolution microscopy revealed the asynchronicity of nuclear division in the blood stage, as depicted in detail in Fig. 1D; in this multinucleated cell, one nucleus has two CEN-4 foci close to each other and another has two CEN-4 foci on opposite sides. Fig. 1De,Df provide two 45° views of early schizonts as can be visualized in Movies 1–5.

The localization of CEN4-GFP was also analyzed with immunofluorescence assays (IFAs) using fixed cells and antibodies to the marker for centrosome-associated *Chlamydomonas* centrin, which also recognizes the human CEN-1 and to alpha-tubulin, known to stain the microtubule network (Mahajan et al., 2008).

There was clear co-localization between CEN4-GFP and the *Chlamydomonas* centrin antibody (Fig. S2A) and, as expected, alpha tubulin was observed as a single focus between two centrin foci (Fig. S2B). The results were consistent with replication and division of the putative MTOC and the nucleus occurring together during schizogony in the liver and blood stage and sporogony in the mosquito vector, as depicted for a blood stage schizont in the schematic (Fig. 1E).

### CEN-4 is not required for parasite proliferation at any stage in the *P. berghei* life cycle

To examine the role of CEN-4 throughout the life cycle, we created a null mutant by deletion of the *cen-4* gene using double homologous recombination (Fig. S3A). Two independent parasite clones were generated: Δ*cen-4* cl.1 and Δ*cen-4* cl.3, and diagnostic PCR confirmed deletion of the *cen-4* gene by integration of the *dhfr/ts* cassette (Fig. S3B, data for clone 1). Southern blot confirmed deletion of the gene (Fig. S3C).

**Fig. 2.**
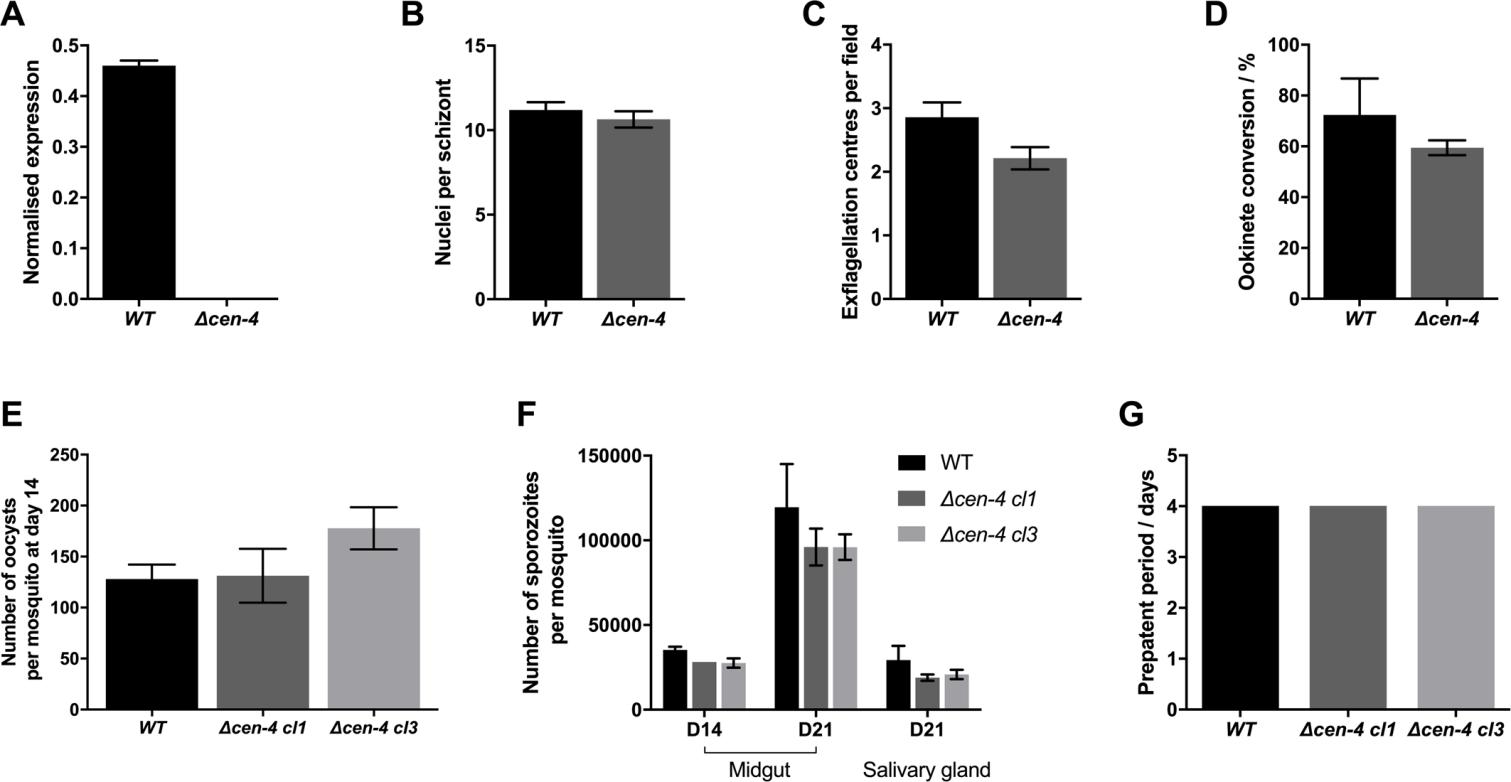
**CEN-4 is not essential throughout the *P. berghei* parasite life cycle.** (A) Quantitive RT-PCR analysis of *cen-4* mRNA levels in asexual blood stage *Δcen-4* and WT parasites. Normalized expression relative to *hsp70* (PBANKA_0818900) and *arginyl-t RNA synthetase* (PBANKA_1434200) genes. Data presented are mean±s.e.m. of three technical replicates and three biological replicates. (B) Number of mature merozoites within *Δcen-4* and WT schizonts. Data presented are average number of nuclei per schizont ±s.e.m. from three biological replicates. (C) Microgametogenesis in Δ*cen-4* and WT parasites measured as the number of exflagellation centres per field. Data presented are average number of exflagellation centres per field ±s.e.m. Three technical replicates and three biological replicates. (D) Percentage of ookinete conversion in Δ*cen-4* and WT parasites. Ookinetes were identified using the antibody marker 13.1 and were defined as cells that had successfully differentiated into elongated ookinetes. Data presented are average number ookinetes ±s.e.m. of three technical replicates and three biological replicates. (E) Number of oocysts per midgut (at 14 dpi) of Δ*cen-4* clones (clones 1 and 3 obtained from two independent transfections) or WT parasite-infected mosquitoes. Data presented are average number of oocysts per 20 mosquito guts counted ±s.e.m. of two independent transfection from two independent clones. (F) The number of sporozoites in a mosquito midgut at 14 and 21 dpi or salivary glands at 21 dpi in Δ*cen-4* clones (clones 1 and 3) or WT parasite-infected mosquitoes. Data presented are average number of oocysts per 20 mosquito guts counted ±s.e.m. of two independent transfection from two independent clones. (G) Bite-back experiments measure day of patent blood stage parasitaemia indicative of transmission from mosquito to mouse. (*n*=3).

Quantitative RT-PCR (qRT-PCR) analysis using mature schizont cDNA was used to confirm the absence of transcript in the *Δcen-4* line (Fig. 2A). Analysis of both *Δcen-4* clones (only clone 1 is shown) showed a similar number of nuclei/developing merozoites in the late blood stage schizont when compared with WT control parasites (Fig. 2B). No difference was observed in *in vitro* microgamete exflagellation or ookinete conversion between mutant and WT parasites (Fig. 2C,D). The number of *Δcen-4* oocysts (representative data from the two different clones) on the *Anopheles stephensi* midgut was not significantly different from the number of WT parasites at 14 dpi (Fig. 2E) and no significant difference was observed in the number of midgut sporozoites at 14 and 21 dpi and salivary gland sporozoites at 21 dpi per mosquito, in comparison to the WT parasite control for the Δ*cen-4* cl1 and cl3 (Fig. 2F). Furthermore, there was no significant difference in sporozoite infectivity in bite-back experiments (Fig. 2G), in which infected mosquitoes were allowed to feed on mice, establishing a blood stage infection following replication in the liver. No significant difference was observed in the level of *cen-1*, *cen-2* and *cen-3* transcripts in the absence of *cen-4* (Fig. S3D), suggesting that over-expression of other centrin family members does not compensate for the loss of CEN-4 function. The *Chlamydomonas* centrin antibody showed a similar signal in both Δ*cen-4* and WT parasites (Fig. S3E).

The GFP-tagged CEN-4 was immunoprecipitated with an anti-GFP antibody from early blood stage schizonts when there are 4–6 nuclei within one cell and the precipitate was analyzed by tryptic digestion and tandem mass spectroscopy to identify any proteins associated with CEN-4 (Fig. S3F). In independent duplicate analyses, peptides from CEN-1, -2 and -3, as well as CEN-4 were detected. We hypothesize that the four centrins could form a protein complex during early blood stages but further analysis would be necessary to confirm this hypothesis.

## DISCUSSION

Closed atypical mitosis during *Plasmodium* schizogony is regulated in part by the putative MTOC, a structure likely analogous to that of the yeast SPB (Arnot et al., 2011; Gerald et al., 2011). We show here by live cell imaging the localization and dynamics of *Pb*CEN4-GFP, a GFP-tagged version of the alveolate-specific centrin during the three phases of asexual replication in *Plasmodium,* which extends previous studies (Mahajan et al., 2008). We delineated and focused on the temporal profile of expression during schizogony in host liver and red blood cells and sporogony on the mosquito midgut. *Pb*CEN4-GFP location in non-dividing parasites (the merozoite in the vertebrate and the sporozoite in the mosquito) is largely cytosolic; but once nuclear replication and division begins, *Pb*CEN4-GFP is concentrated at the putative MTOC as described previously for *P. falciparum* CEN-2 and -3 (Mahajan et al., 2008). Although nuclear division within the liver and blood stages is largely similar, some differences are apparent. In the liver, for example, when the nuclei start to replicate and divide, CEN-4 is mainly cytosolic, whereas at this point in blood stages, CEN-4 is already at specific foci. *Pb*CEN-4 has a location similar to that of other centrins in model systems such as yeast and mammalian cells, which is in association with the MTOC (Paoletti et al., 1996). Moreover, an additional diffuse location in the cytoplasm has been described previously (Paoletti et al., 1996). It is possible that CEN-4 is stored in the cytosol in preparation for mitosis, prior to concentration at the putative MTOC, an accumulation which may reflect the relatively high rate of nuclear replication compared to that in other organisms such as mammalian cells for example; during schizogony each nucleus divides approximately every 2 h in the liver stage and every 5 h in the blood stage (M.R. and R.R.S., unpublished).

While the deletion of *cen-4* does not affect parasite development, we were unable to delete *cen*-*1*, *-2* or *-3*, indicating that these three centrins are likely essential for asexual blood stage development, and consistent with a role in MTOC duplication, as found in other lower eukaryotes such as *S. cerevisiae* where CDC31 is essential for SPB duplication (Spang et al., 1993). It is only its localization and its complex formation with the other centrins that implicates CEN-4 in MTOC biology, and it is possible that this alveolate-specific centrin may have other roles in cell function during the life cycle. For example, in a triple centrin-deficient (Cen4-/Cen3-/Cen2-) chicken cell line, cell viability and proliferation were not affected, but this variant cell is less efficient in repairing UV-induced DNA damage (Dantas et al., 2011).

The fact that peptides of CEN-1, CEN-2 and CEN-3 were detected when CEN4-GFP was immunoprecipated allowed us to hypothesize that the four centrins may form a protein complex during blood stage development, although this remains to be proven. Further investigations would be required to establish whether the hypothesized interactions are direct or indirect and whether or not this hypothetical complex may be part of a larger complex together with other proteins.

For example, does the absence of CEN-4 affect the stoichiometry of the other centrins and/or are other proteins recruited to the hypothetic complex? It may be possible to answer this question if smaller epitope tags can be added to the other centrins or specific antibodies developed to facilitate purification of the complex.

## MATERIALS AND METHODS

### Ethics statement

All animal work performed in the UK passed an ethical review process and was approved by the United Kingdom Home Office. Work was carried out under UK Home Office Project Licenses (40/3344 and 30/3248) in accordance with the United Kingdom ‘Animals (Scientific Procedures) Act 1986’ and in compliance with ‘European Directive 86/609/EEC’ for the protection of animals used for experimental purposes. Experiments performed in Switzerland were conducted in strict accordance with the guidelines of the Swiss Tierschutzgesetz (TSchG; Animal Rights Laws) and approved by the ethical committee of the University of Bern (Permit Number: BE109/13). Six-to-eight week old female Tuck-Ordinary (TO) (Harlan) outbred mice were used for all experiments in the UK. Balb/c female mice between six and ten weeks of age were used in experiments in Switzerland. Mice were either bred in the central animal facility of the University of Bern, or were supplied by Harlan Laboratories or Charles River Laboratories.

### Generation of transgenic parasites

For GFP-tagging of *cen-4* by single homologous recombination, a 599 bp region of *cen-4* (PBANKA_0941400) starting 70 bp downstream of the ATG start codon and omitting the stop codon was amplified using primers T1671 and T1672. This DNA fragment was inserted using *Kpn*I and *Apa*I restriction sites upstream of the *gfp* sequence in the pOB277 plasmid containing a human *dhfr* cassette to confer resistance to pyrimethamine. The vector was linearized with *Hin*dIII before transfection*.* The Δ*cen4* gene-knockout targeting vector was constructed using the pBS-DHFR plasmid, which contains polylinker sites flanking a *T. gondii dhfr/ts* expression cassette, as described previously (Tewari et al., 2010). A 628 bp fragment at the 5′ end of the *cen-4* sequence was generated from genomic DNA using PCR primers N0941 and N0942 and inserted into pBS-DHFR using *Apa*I and *Hin*dIII restriction sites upstream of the *dhfr/ts* cassette. A 676 bp fragment generated with primers N0943 and N0944 from the 3′ region of *cen-4* was then inserted downstream of the *dhfr/ts* cassette using *Eco*RI and *Xba*I restriction sites. The linear targeting sequence was released using *Apa*I/*Xba*I digestion of the plasmid.

The sequences of the oligonucleotides used to make these constructs are presented in Table S1. *P. berghei* ANKA line 2.34 (for GFP-tagging) or ANKA line 507cl1 (for gene deletion) were transfected by electroporation (Janse et al., 2006). Briefly, electroporated parasites were mixed immediately with 100 μl of reticulocyte-rich blood from a naïve mouse treated with phenylhydrazine (6 mg/ml) (Sigma-Aldrich), incubated at 37°C for 20 min and then injected intraperitoneally into another mouse. From day 1 post infection, pyrimethamine (70 μg/ml) (Sigma-Aldrich) was supplied in the drinking water for 4 days. Mice were monitored for the appearance of parasites for 15 days and drug-resistant parasites were passaged into a second mouse with continuing drug selection. Parasites were then cloned by limiting dilution and subsequently genotyped.

### Parasite genotype analysis

For the parasites expressing a C-terminal GFP-tagged CEN-4 protein, diagnostic PCR was used with primer 1 (IntT167) and primer 2 (ol492) to confirm integration of the GFP targeting construct.

For the gene knockout parasites, diagnostic PCR was used with primer 1 (IntN094) and primer 2 (ol248) to confirm integration of the targeting construct, and primer 3 (N094KO1) and primer 4 (N094KO2) were used to confirm deletion of the *cen4* gene.

For Southern blotting, genomic DNA from WT and mutant parasites was digested with *Pac*I and separated on a 0.8% agarose gel before blotting onto a nylon membrane (GE Healthcare). A probe was generated from a PCR fragment homologous to the 3′ region just outside of the targeted region using the AlkPhos direct labelling kit (GE Healthcare) according to manufacturer's instructions.

### Parasite phenotype analysis

Gametocyte exflagellation was examined on day 4–5 post-infection (Billker et al., 1998): after 15 min in ookinete culture medium, exflagellation centres were counted by phase contrast microscopy. Ookinete formation was assessed after 24 h: cultured cells were pelleted for 2 min at 2300 ***g*** and then resuspended and incubated with 50 μl of ookinete medium containing Hoechst 33342 DNA dye at a final concentration of 5 μg/ml and a Cy3-conjugated mouse monoclonal antibody 13.1 that binds to the P28 protein on the surface of ookinetes and any undifferentiated macrogametes or zygotes (Tewari et al., 2005). P28-positive cells were counted with a Zeiss AxioImager M2 microscope (Carl Zeiss, Inc) fitted with an AxioCam ICc1 digital camera. Ookinete conversion was expressed as the percentage of P28-positive parasites that differentiated into fully mature (stage 6) ookinetes (Janse et al., 1986). For mosquito transmission experiments, 20–50 female *Anopheles stephensi* SD500 mosquitoes were allowed to feed for 20 min on anaesthetized infected mice with an asexual parasitaemia of ∼12–15% and comparable numbers of gametocytes as determined from Giemsa-stained blood films. At 14 dpi, 20 mosquitoes were dissected and the midgut oocysts were counted: oocysts were stained with Hoechst 33342 in PBS for 10–15 min and the guts were mounted under Vaseline-rimmed cover slips to allow counts and images to be recorded using 10× and 63×oil immersion objectives on a Zeiss AxioImager M2 microscope fitted with an AxioCam ICc1 digital camera. The same mosquito midgut samples were disrupted in a loosely fitting homogenizer to release sporozoites, which were then quantified using a haemocytometer. For 21 dpi mosquitoes, salivary glands and midguts were dissected and disrupted in a loosely fitting homogenizer to release sporozoites, which were then quantified. Mosquitoes infected with WT or Δ*cen4* parasites were used to transmit parasites back to TO mice in bite-back experiments, measuring blood stage parasitemia by light microscopy of Giemsa-stained blood films slides four days after feeding.

### Quantitative RT-PCR

Parasites were purified from schizonts and the RNA was isolated using the Absolutely RNA purification kit (Stratagene). cDNA was synthesized using an RNA-to-cDNA kit (Applied Biosystems). Gene expression was quantified using 250 ng of total RNA and qPCR reactions were prepared with 2 µl of cDNA, 5 µl SYBR green fast master mix (Applied Biosystems), 0.5 µl (250 nM) each of the forward and reverse primers and 2 µl DEPC-treated water. Primers were designed using primer3 software (Primer-blast, NCBI), and amplified a region of 321 bp for *cen-1*, 260 bp for *cen-2*, 141 bp for *cen-3* and 82 bp for *cen-4*. Analysis was performed on an Applied Biosystems 7500 fast machine with the following cycling conditions: 95°C for 20 s followed by 40 cycles of 95°C for 3 s; and 60°C for 30 s. Three technical replicates and three biological replicates were performed for each assayed gene. The *hsp70* (PBANKA_0818900) and *arginyl-t RNA synthetase* (PBANKA_1434200) genes were used as endogenous control reference genes. The primers used for qPCR analysis of all centrins can be found in Table S1.

### Western blot and immunoprecipitation analysis

Parasite-infected red blood cells were placed in schizont culture medium (RPMI 1640, FCS 1:10, Penicillin/Streptomycin 1:100) for 24 h at 37°C and parasites were allowed to mature to schizonts, which were purified the following day on a 60% v/v NycoDenz (in PBS) gradient, harvested from the interface and washed (stock solution: 27.6% w/v NycoDenz in 5 mM Tris-HCl, pH 7.20, 3 mM KCl, 0.3 mM EDTA). After the addition of Laemmli sample buffer to the cells, the samples were boiled and electrophoresed on a 4–12% SDS-polyacrylamide gel. Resolved proteins were subsequently transferred to nitrocellulose membranes (Amersham Biosciences) and immunoblotting performed using the Western Breeze Chemiluminescent Anti-Rabbit kit (Invitrogen) and anti-GFP polyclonal antibody (Invitrogen) at a concentration of 1:1250, according to the manufacturer's instructions.

A schizont lysate was also used for immunoprecipitation with a GFP-Trap^®^_A Kit (Chromotek) following the manufacturer's instructions. The GFP-Trap^®^_A beads were equilibrated with dilution buffer and proteins bound from the parasite lysate by slow mixing at 4°C for 2 h. The beads were then harvested by centrifugation at 1200 ***g*** for 2 min and washed twice with dilution buffer. The beads with bound proteins were then treated with trypsin and released peptides were analyzed by tandem mass spectrometry.

### Liver stage parasite imaging

For *P. berghei* liver stage parasites, 100,000 HeLa cells were seeded in glass-bottomed imaging dishes. Salivary glands of female *A. stephensi* mosquitoes infected with CEN4-GFP parasites were isolated and disrupted using a pestle to release sporozoites, which were pipetted gently onto the seeded HeLa cells and incubated at 37°C in 5% CO_2_ in complete minimum Eagle's medium containing 2.5 μg/ml amphotericin B (PAA). Medium was changed 3 h after initial infection and once a day thereafter. For live cell imaging, Hoechst 33342 (Molecular Probes) was added to a final concentration of 1 μg/ml, and parasites were imaged at 24, 48, 55 h post-infection using a Leica TCS SP8 confocal microscope with the HC PL APO 63×/1.40 oil objective and the Leica Application Suite X software.

### Indirect immunofluorescence assay

IFAs for *P. berghei* were performed on poly-L-lysine coated slides where schizonts had been previously fixed in 2% paraformaldehyde (PFA) in MTSB (10 mM MES, 150 mM NaCl, 5 mM EGTA, 5 mM MgCl_2_, 5 mM glucose) in PBS 1× for 30 min at RT and smeared onto slides. The fixed cells were permeabilized with TBS containing 0.2% Triton X-100 for 5 min and washed three times with TBS before blocking. Blocking was performed using 3% BSA (w/v), 10% goat serum (v/v) in TBS for 1 h at RT and TBS containing 1% BSA and 1% goat serum was used to dilute the antibodies for the incubations. Anti-GFP rabbit antibody (Invitrogen) was used at a 1:250 dilution, anti-alpha-tubulin mouse antibody (Sigma-Aldrich) was used at a 1:1000 dilution, and anti-centrin mouse clone 20h5 antibody (Millipore) was used at a 1:300 dilution; each was incubated for 1 h at RT. Three washes were performed with TBS, then AlexaFluor 488 labelled anti-rabbit (green) and AlexaFluor 568 labelled anti-mouse (red) (Invitrogen) (1∶1000 dilution) were used as secondary antibodies and incubated for 1 h at RT. Slides were mounted with vectashield containing DAPI (blue) and sealed with nail polish. *P. berghei* images were captured as described for live imaging using a 63×oil immersion objective. Control images using a WT parasite line that does not express GFP were also acquired using the same methodology to assess background signal.

### Super resolution microscopy

Cells were fixed with 4% PFA at room temperature, washed and then the DNA was stained with DAPI. Samples were stored at 4°C in PBS, until imaging on the same or the following day. 4.5 µl of cell suspension was mounted under a high precision coverslip (Zeiss 1.5H, 474030-9000-000); the coverslip edges were sealed with a minimal amount of silicon grease or with CoverGrip™ (Biotium, cat. no. 23005) to avoid evaporation and then scanned with a Zeiss C-Apochromat 63×/1.2 W Korr M27 water immersion objective on a Zeiss Elyra PS.1 microscope, using the structured illumination microscopy (SIM) technique. The correction collar of the objective was set to 0.17 to achieve optimum contrast. The following settings were used: lasers, 405 nm: 15%, 488 nm: 8%; exposure times, 150 ms, five grid rotations, five phases. The bandpass filters BP 420-480+LP 750 and BP 495-550+LP 750 were used for the blue and green channels, respectively. Multiple focal planes (Z stacks) were recorded. Images were processed and maximum projections or Z stacks were visualized with the 3D visualization module. Processing and export of images and videos were done by Zeiss Zen Black 2012 Service Pack 5 or Volocity. Z-stacks were visualized either as maximum projections in 2D or visualized by the 3D rendering module of Zeiss Zen Black, or Volocity. The images recorded in multiple focal planes (Z-stack) were 3D rendered into virtual models and exported as movies.

## Supplementary Material

Supplementary information
