# Supplementary material for: Plasmodium centrin PbCEN-4 localizes to the putative MTOC and is dispensable for malaria parasite proliferation
Source: Biol Open. 2018 Dec 12;8(1):bio036822. doi: 10.1242/bio.036822 (PMC6361220; doi:10.1242/bio.036822)
Supplement: Supplementary information [file biolopen-8-036822-s1.pdf]

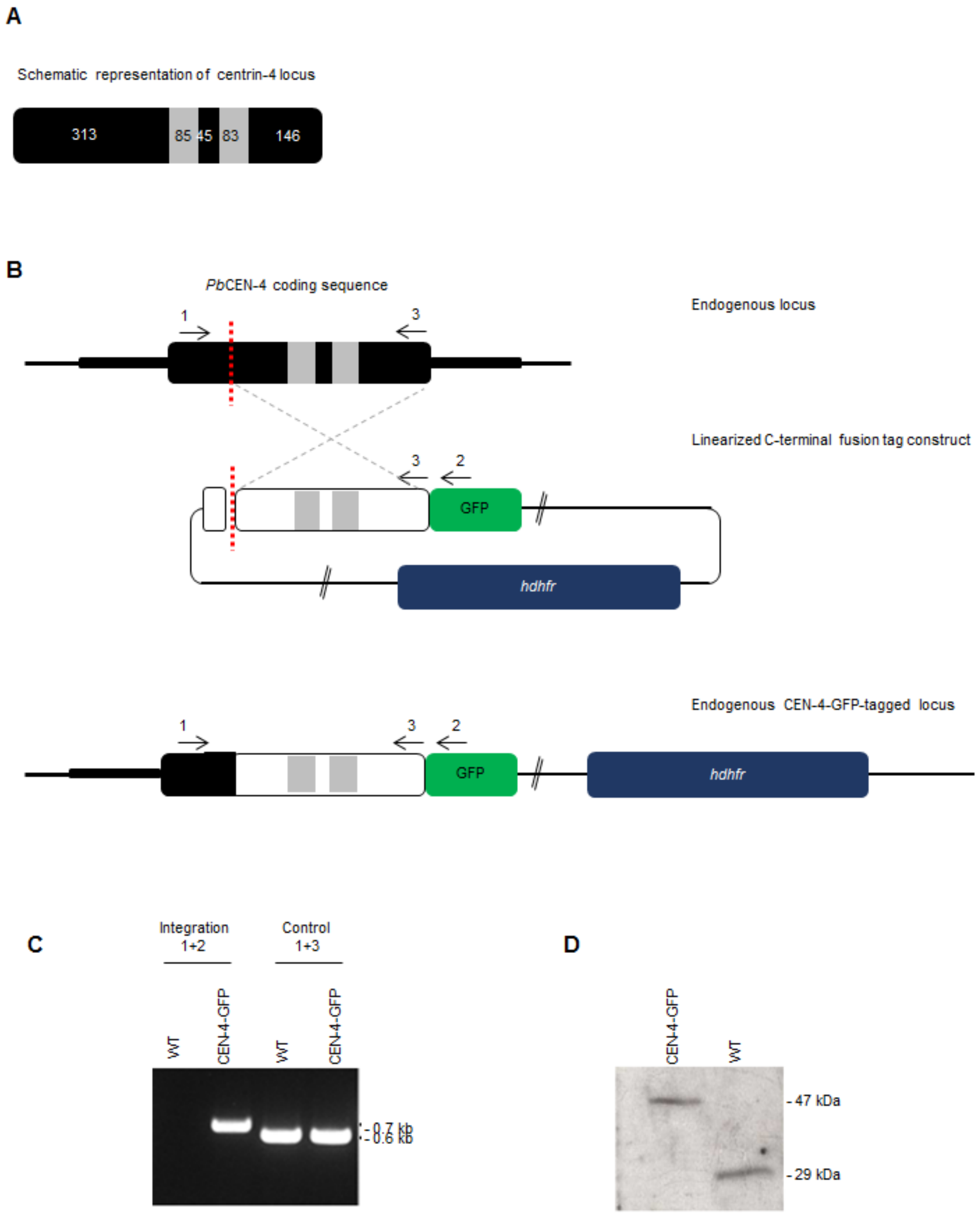

Biology Open • Supplementary information

Figure S1. Generation and genotypic analysis of a CEN-4-GFP parasite line.

A) Schematic representation of the *cen-4* locus, representing exons as black boxes and introns as grey boxes. The centrin-4 gene possesses three exons with a length of 313, 45 and 146 base pairs and two introns with a length of 85 and 83 base pairs.

B) Schematic representation of cen-4 locus, the GFP-tagging construct and the recombined *cen-4* locus following single homologous recombination. The PCR primers used to confirm successful integration of the construct are indicated by arrows 1 and 2 and those used for the PCR control are indicated by arrows 1 and 3. The red dashed line represents the linearization site *Hind*III.

C) Diagnostic PCR of CEN-4-GFP and WT parasites using primers IntT167 (Arrow 1) and ol492 (Arrow 2). Integration of *the cen-4* tagging construct produces a 0.7 kb band. The control PCR is performed by using IntT167 (Arrow 1) and the reverse primer used to amplify the cloned region T1672 (Arrow3). CEN-4-GFP = parasite line

D) Western blot of lysates from blood stage parasites expressing either CEN-4-GFP (47 kDa) or WT-GFP (29 kDa), using GFP-Trap antibodies.

Biology Open • Supplementary information

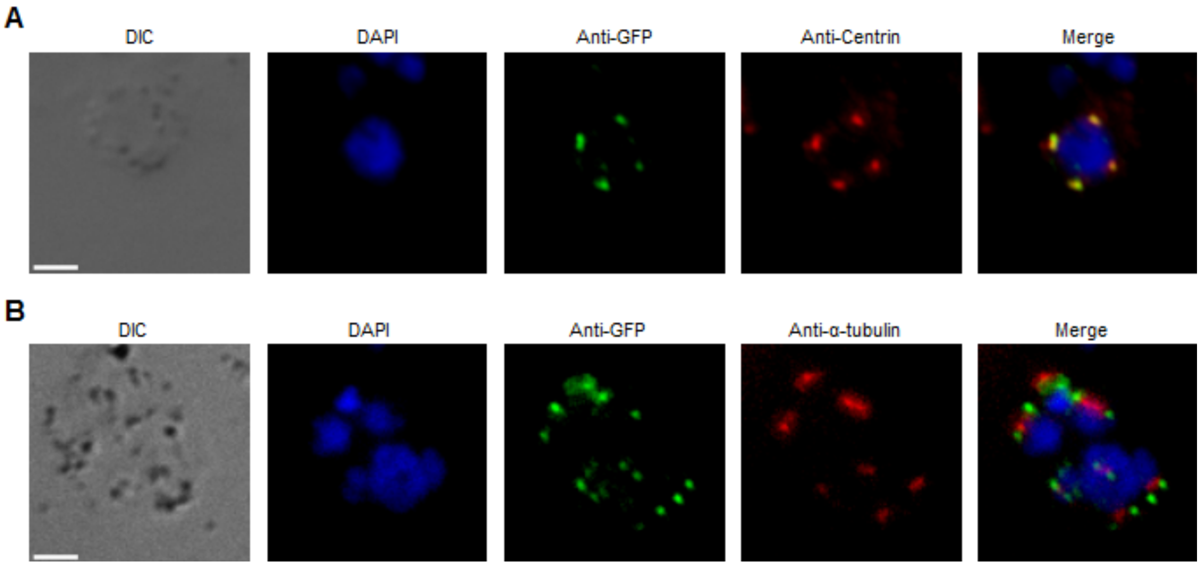

Figure S2. Colocalization IFA assays in CEN-4-GFP parasites

A) Fixed immunofluorescence imaging using anti-GFP and anti-centrin antibodies in early schizonts. CEN-4 shows colocalisation with CEN-1. Scale bar = 5μm

B) Fixed immunofluorescence imaging using anti-GFP and anti-alpha-tubulin antibodies in early schizonts. In dividing nuclei, CEN-4 is present as two individual foci, which lie either side of an alpha-tubulin focus. Scale bar = 5 μm

Biology Open • Supplementary information

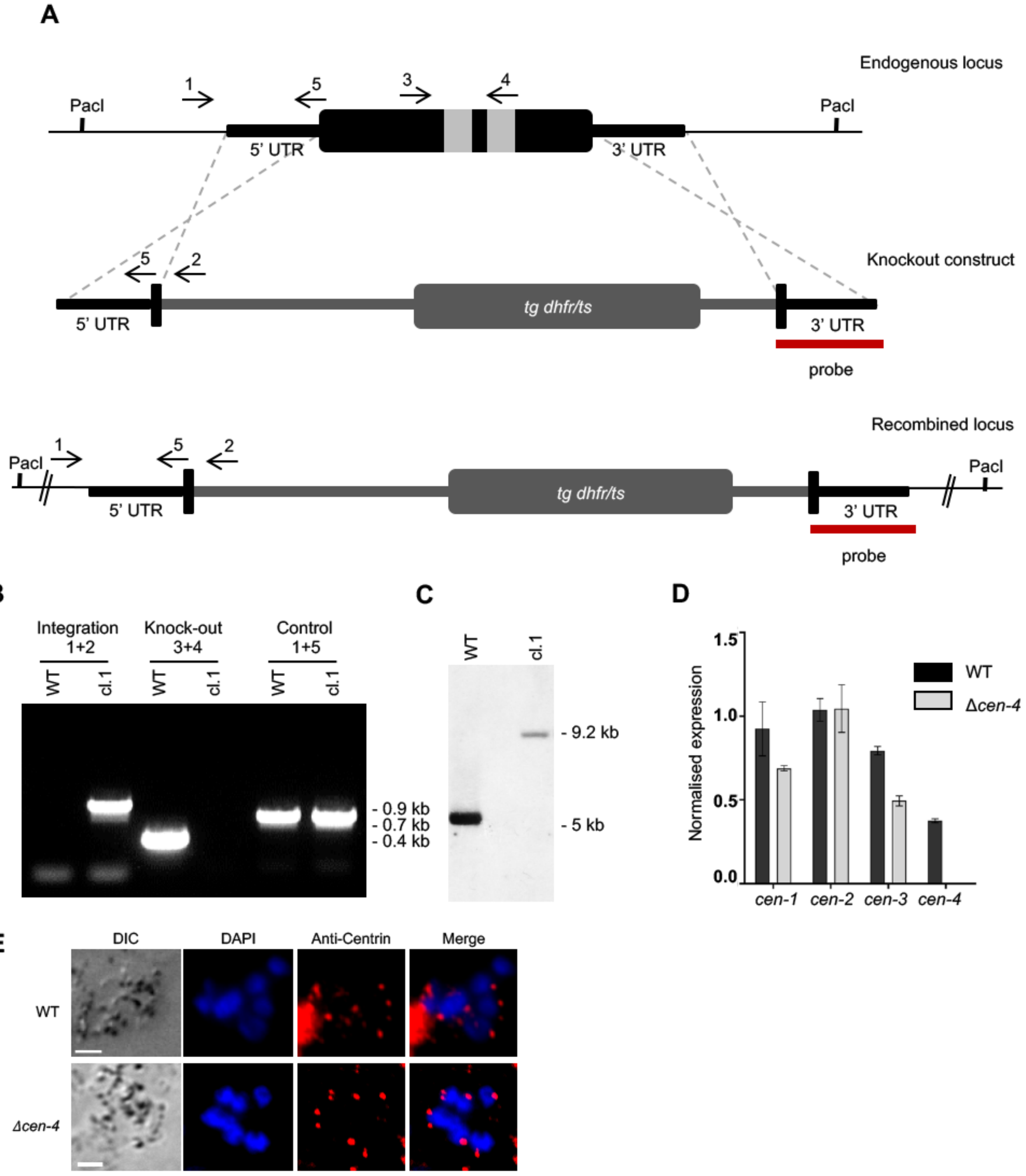

F

Accession number	Protein name	Molecular weight (kDa)	Number of peptides
PBANKA_0206300	Centrin-1 (CEN-1)	20 kDa	5
PBANKA_1310400	Centrin-2 (CEN-2)	19 kDa	7
PBANKA_0511800	Centrin-3 (CEN-3)	21 kDa	5
PBANKA_0941400	Centrin-4 (CEN-4)	20 kDa	26

Fig. S3. Generation and genotypic analysis of the *Δcen-4* parasite line.

A) Schematic representation of the endogenous *cen-4* locus, the gene deletion targeting construct and the recombined *cen-4* locus following double homologous recombination. PCR primers used to confirm successful integration of the construct are indicated by arrows 1 and 2 and PCR primers used to confirm deletion of the *cen-4* gene are indicated by arrows 3 and 4 in both clones. The combination of primers indicated by arrows 1 and 5 is used as a control PCR. The Southern probe hybridizes the *cen-4* 3' UTR region is represented in red on the schematic.

B) Diagnostic PCR analysis of the *cen-4* locus in *Δcen-4* and WT parasites using primers IntN094 (arrow 1) and ol248 (arrow 2). Integration (Integration) of the targeting construct results in a band of 0.9 kb, while PCRs amplifying the gene (Knock-out) produce a band of 0.4 kb (arrows 3 and 4; primers N094KO1 and -2). The control PCR (Control) is performed by using primers IntN094 (arrow 1) and N0942 (arrow 5).

C) Southern blot analysis of *Δcen-4* and WT parasite genomic DNA. A probe specific for the *cen-4* 3'UTR hybridized to a 5 kb fragment from WT, and a 9.2 kb fragment from *Δcen-4* parasites.

D) Quantitive RT-qPCR analysis of *cen-1*, *cen-2*, *cen-3* and *cen-4* mRNA levels in asexual blood stage *Δcen-4* and WT parasites. Data presented is mean ±SEM of 3 technical replicates and 3 biological replicates.

E) Fixed immunofluorescence imaging using the *Chlamydomonas* centrin antibody in early schizonts in the *Δcen-4* and WT parasite lines. In dividing nuclei, in the absence of CEN-4, the antibody possesses the same signal as in CEN-4-GFP parasites. Scale bar = 5 µm

F) Centrins identified amongst proteins immunoprecipitated with anti-GFP antibodies from lysates of early blood schizonts of CEN-4 GFP-expressing parasites. The gene and protein names and the predicted protein size, as well as the number of peptides identified, are shown.

Biology Open • Supplementary information

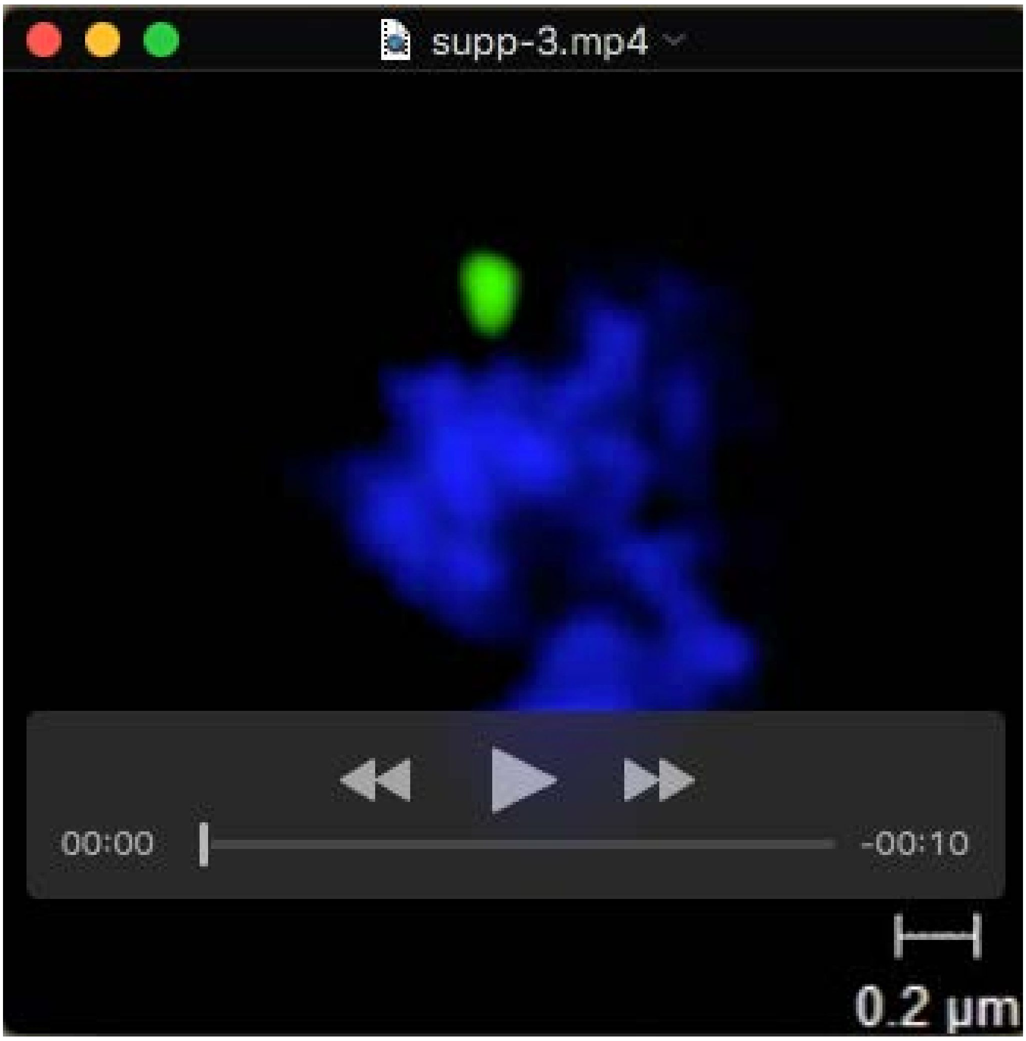

Movie 1. Fixed asexual blood stage parasites showing one CEN-4 focus shown by 3D rendered super resolution microscopy (3D SIM).

Biology Open • Supplementary information

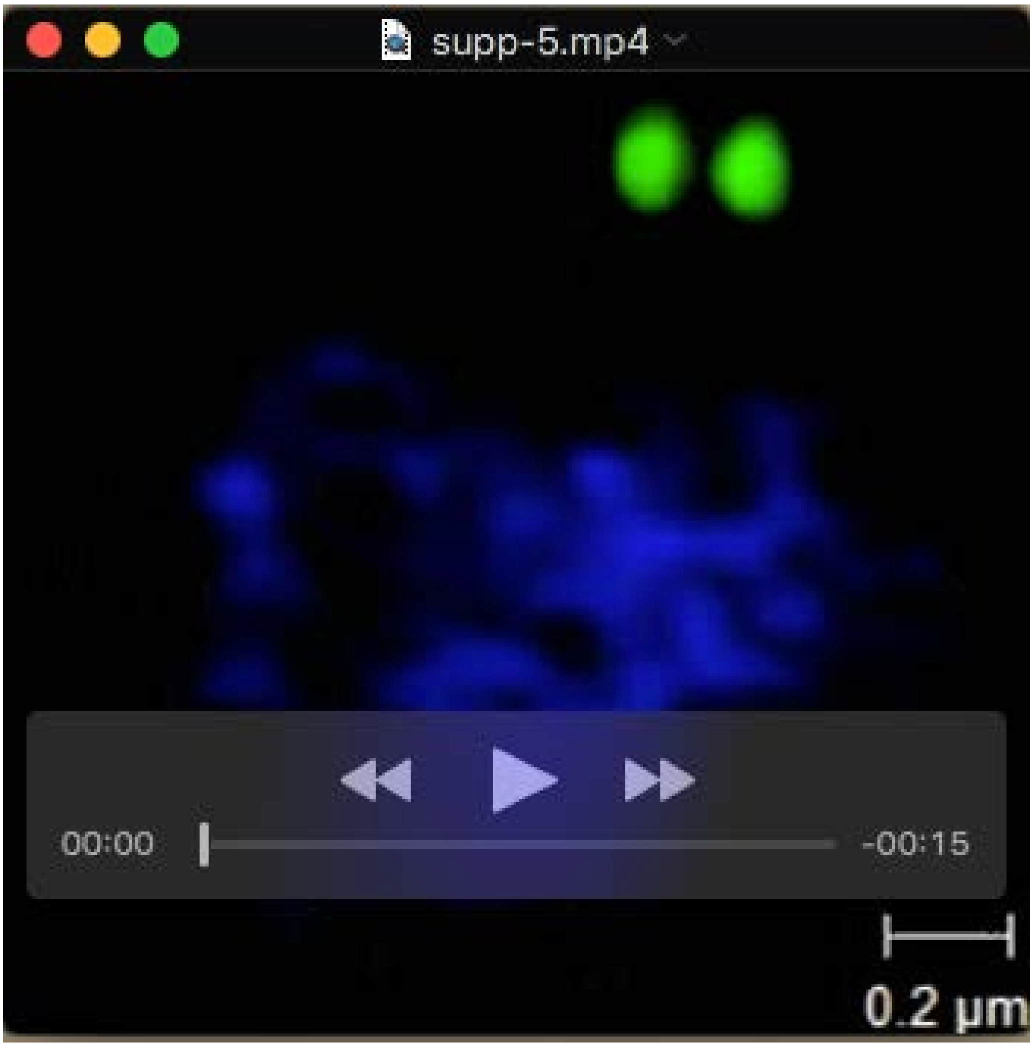

Movie 2. Fixed asexual blood stage parasites showing duplicated CEN-4 foci shown by 3D rendered super resolution microscopy (3D SIM).

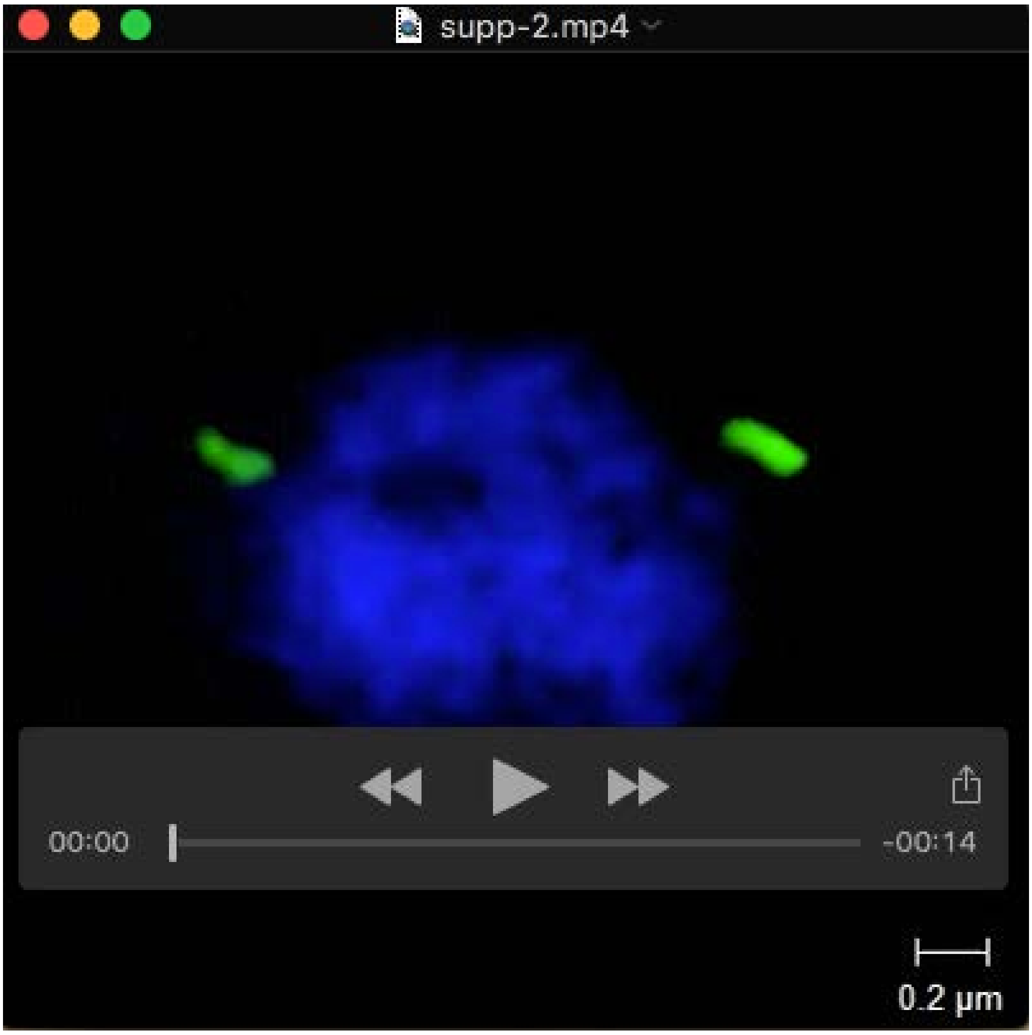

Movie 3. Fixed asexual blood stage parasites showing two segregated CEN-4 foci shown by 3D rendered super resolution microscopy (3D SIM).

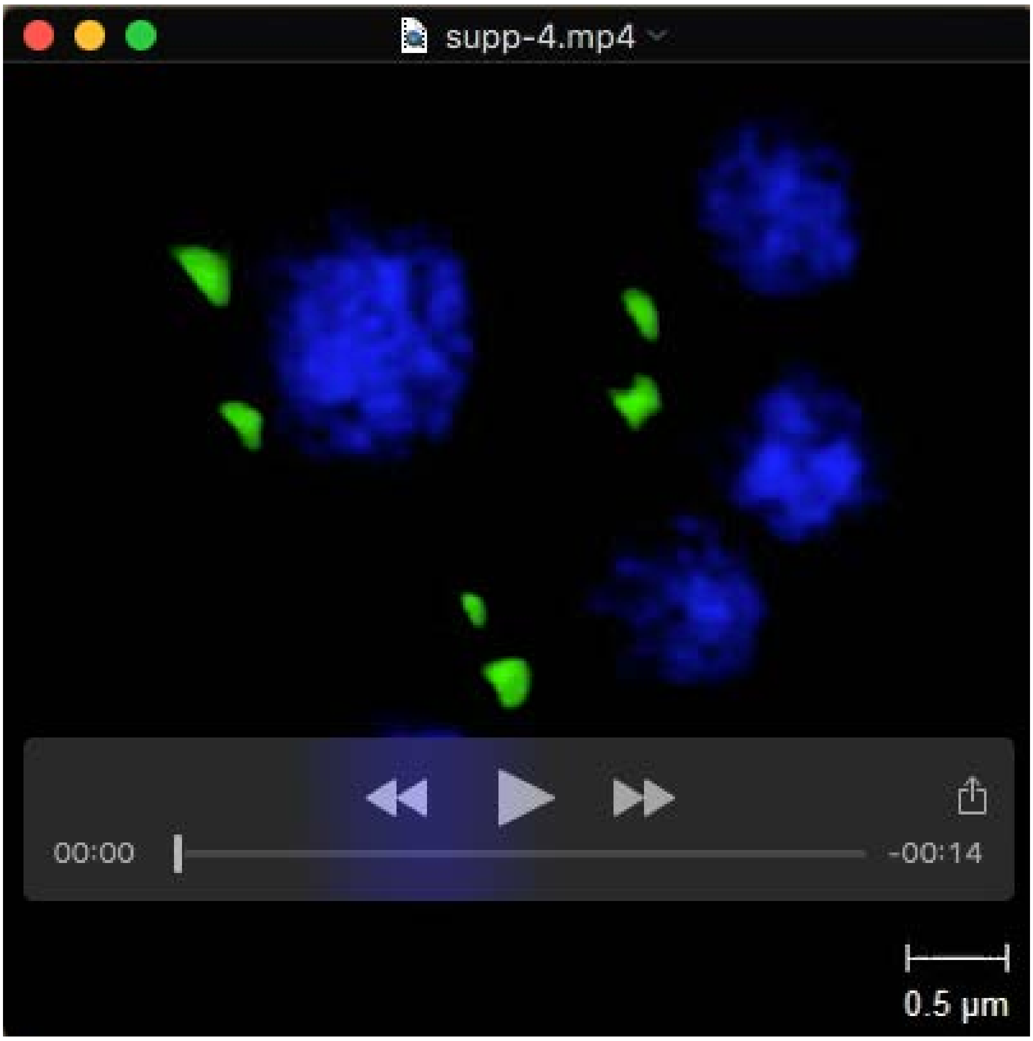

Movie 4. Asynchronicity of nuclear division in a single blood stage schizont, shown by 3D rendered super resolution microscopy (3D SIM).

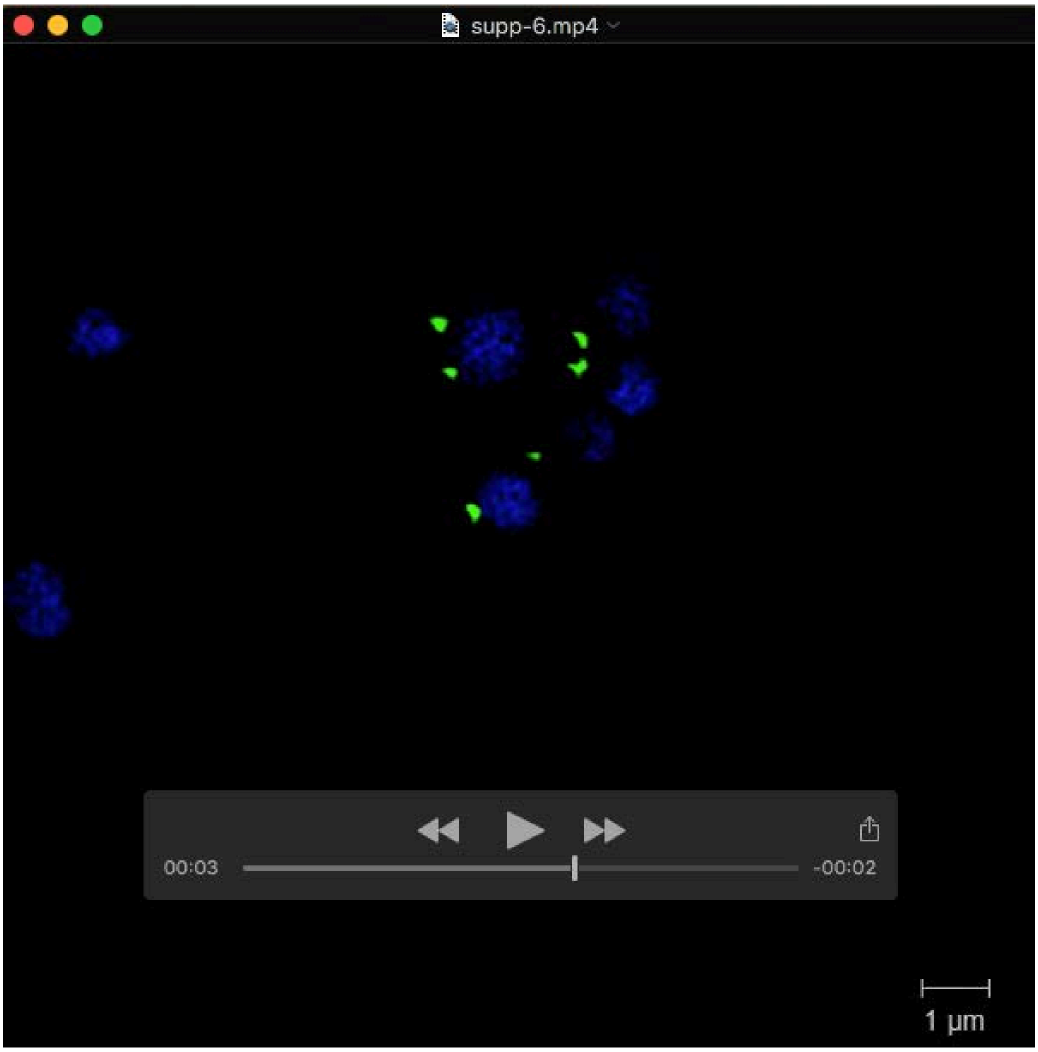

Movie 5. Asynchronicity of nuclear division in a single blood stage schizont, all focal planes are presented sequentially (Z focus stack).

Biology Open • Supplementary information
